# Functional Outcomes of Fluoroscopy-Guided Intra-articular Steroids in Lumbar Facet Arthropathy: A Retrospective Comparative Study of Dexamethasone Versus Triamcinolone Acetonide

**DOI:** 10.7759/cureus.61551

**Published:** 2024-06-02

**Authors:** Gowtham Gandhi, Prabhu Ethiraj, Manoj K Ramachandraiah, Arun Kumaar

**Affiliations:** 1 Department of Orthopaedics, Sri Devaraj Urs Medical College, Sri Devaraj Urs Academy of Higher Education and Research, Kolar, IND

**Keywords:** triamcinolone acetonide, dexamethasone, transforaminal epidural steroid injection, nerve root block, low back ache

## Abstract

Introduction

Mechanical low back pain frequently originates from the lumbar facet joint (LFJ). Axial low back discomfort can result from osteoarthritis in the LFJ. Depending on the severity of LFJ degeneration, the effect of intra-articular (IA) LFJ corticosteroid injection may vary. For LFJ discomfort, IA block with steroids and local anaesthetics has also been utilised, with varying degrees of success. The main objective of this study was to assess the efficacy of IA steroid injections dexamethasone vs. triamcinolone acetonide for the treatment of LFJ syndrome and to compare functional outcome in terms of Visual Analog Scale (VAS) score, Modified Oswestry Disability Index (MODI) score, and short-form McGill Pain Questionnaire between the two groups.

Methodology

Dexamethasone 8 mg or triamcinolone acetonide 40 mg was given intra-articularly to 27 patients comprising group A and 33 patients comprising group B, respectively (total 60 patients). Before intervention and at one, three, and six months, observation was conducted using the VAS score, short-form McGill pain questionnaire, and MODI score.

Results

There was a significant difference between both the groups after the procedure with pain alleviation and functional improvement, more in the group that received triamcinolone acetonide. A significant difference was observed in all three parameters that assessed pain with differences more pronounced at six months.

Conclusion

Pain reduction and clinical outcomes were better among the group that received triamcinolone acetonide. Injection of a steroid alone is associated with its own side effects. When a lumbar transforaminal epidural injection is used to treat radiculopathy in the lumbar area, particulate medication (triamcinolone) is more effective than non-particulate medication (dexamethasone) with no known drug-related complications.

## Introduction

Chronic low back pain (CLBP) has been identified as a cause of absence from work and disability [1]. The pooled point prevalence of low back pain in India was 48%, while the annual prevalence was 51%, and the lifetime prevalence was 66%. The combined prevalence rates were higher among females, people living in rural areas, and elementary school employees [2]. LBP is the second-leading cause of activity restriction and work restriction in most of the world, after upper respiratory illnesses. LBP is currently the primary cause of disability worldwide [3]. Mechanical low back discomfort and pain frequently originate from the lumbar facet joint (LFJ). The facet joint is the posterolateral articulation that connects one vertebra's inferior articular process to the superior articular process of the vertebra below. The facet joints aid in posterior load bearing, and spine stability during flexion and extension, and prevent excessive axial rotation. They also play a significant role in load transmission.

Pain radiating into the lower extremity may be experienced when nerve roots leaving the spinal column undergo injury. The term for this is lumbar radiculopathy. The failure of the synovial facet joints is a pathological process known as facet joint arthrosis [4]. Structures close to the LFJ capsule and inside it are innervated by pain-sensitive nerves. Axial low back discomfort can result from osteoarthritis in the LFJ [1]. Nitric oxide, interleukin-6, prostaglandin E2, and certain matrix metalloproteinases are all produced in greater numbers on their own in herniated cervical and lumbar discs. These findings imply that these biochemical substances are connected to the intervertebral disc's degenerative processes [5]. The conservative, interventional, and surgical management of lumbar facet joint syndrome are now the three main therapeutic modalities [6].

Radicular discomfort is treated by transforaminal corticosteroid injections. Steroid injections are believed to be crucial in reducing inflammation around the injured nerve tissue, which therefore reduces pain [7]. However, the additional and even more frequent hazards of epidural/transforaminal injections should also be taken into consideration. These risks include worsening neurological conditions like paralysis and quadriplegia, intravascular injections, cerebrospinal fluid (CSF) fistulas, persistent positional headaches, arachnoiditis, hydrocephalus, air embolism, urinary retention, allergic reactions, stroke, blindness, neurological deficits or paralysis, hematomas, seizures, and death [8]. There are several uncommon, potentially fatal neurologic complications, such as infarctions of the brain and spinal cord [9].

Intra-articular (IA) facet corticosteroid injections (IFSIs) are successful in treating joint radiculopathy brought on by disk herniation and/or spondylosis. When the baseline pain intensity score was low to moderate, IFSIs were more or at least as effective as transforaminal epidural steroid injections (TFESIs) at relieving pain, whereas the comparison between the therapies remained equivocal for severe baseline pain levels. IFSI can serve as a reliable and safer substitute for TFESI because, to our knowledge, there have been no major issues with this method reported yet [10]. IA corticosteroid injections are often and traditionally used to treat facet joint-origin pain and their beneficial effects have been documented in several prior research studies [11].

Depending on the severity of LFJ degeneration, the effect of IA LFJ corticosteroid injection may vary [12]. For LFJ discomfort, IA block with steroids and local anaesthetics has also been used, with varying degrees of success. Steroids possess anti-inflammatory and immunosuppressive properties primarily because of their ability to hinder phospholipase A2 action. The long-term reduction in LBP following facet IA steroid injection, however, might be anywhere between 18% and 63% [13].

Despite the long history of steroid usage in cases of facet arthropathy, in the scientific literature, there is not much evidence to prove which steroid is better amongst dexamethasone and triamcinolone. The objectives of the study were to assess the efficacy of IA steroid injections dexamethasone vs. triamcinolone acetonide to treat LFJ syndrome and to compare functional outcomes in terms of Visual Analog Scale (VAS) score, Modified Oswestry Disability Index (MODI) score, and McGill Pain Questionnaire between the two groups.

## Materials and methods

Study setting

The retrospective hospital-based study was conducted in the orthopaedics department of R.L. Jalapa Hospital and Research Center, Kolar, Karnataka, India, after getting unanimous approval from the institutional ethics committee of Sri Devaraj Urs Medical College, Kolar, Karnataka, India, (No.: SDUMC/KLR/IEC/533/2023-24). Patients who met the inclusion criteria were included in the study.

Study duration

The study period was from September 2022 to February 2023.

Inclusion criteria

Participants needed to be ≥18 years of age. They should have had back pain for at least six months. They should have been able to understand the study protocol, provide voluntary written informed consent, and take part in outcome measurements. Patients who exhibited clinical signs and symptoms consistent with LFJ degenerative changes on lumbar spine radiographs with physiotherapy and other conservative treatments having failed to relieve the persistent pain that had remained for more than one month were included.

Exclusion criteria

The exclusion criteria included patients who had a previous history of spine surgery and a history of malignancies, patients who were diagnosed with lumbar spinal stenosis, spinal instabilities, or vertebral fractures, patients with symptomatic lower limb radiculopathies, and patients with a history of adverse reactions to corticosteroids.

Data collection procedure

A complete history, clinical examination, and radiographs of the lumbosacral spine in the anteroposterior and lateral views were used to evaluate each patient once they met the inclusion and exclusion criteria.

Based on a random number table produced by a computer, patients were divided at random into two groups. Group A comprised 27 participants who were administered dexamethasone (8 mg) IA injection. Group B comprised 33 participants who were administered triamcinolone acetate (40 mg) IA injection.

Fluoroscopically guided transforaminal dexamethasone 8 mg epidural steroid injection was administered to group A (27 participants). In Group B (33 participants), 40 mg of triamcinolone acetate was injected into the epidural space under fluoroscopy guidance. Written informed consent was taken. The VAS and short-form McGill Pain Questionnaire were used to record the baseline pain score before epidural injection, and the MODI was used to assess functional abilities.

Intervention

Patients who met the requirements to be included in the study and who had been nil per oral for eight hours were allowed in the surgery room. A normal saline/ringer lactate fluid infusion was begun at a rate of 15-20 ml/kg. All the standard monitors were attached to a patient, and baseline values were taken. Heart rate, respiration rate, systolic and diastolic blood pressures, mean arterial pressure, saturated percentage of haemoglobin oxygen (SPO2) with pulse oximetry, and electrocardiogram were all monitored as part of standard anaesthesia care.

All the procedures were performed by a single surgeon who is an orthopaedician. He carried out a systematic technique of injectable therapy. The injection locations were marked while being monitored via a fluoroscope. Prior to administering an epidural injection, baseline VAS, MODI, and short-form McGill Pain Questionnaire scores were recorded. Local infiltration lignocaine 1% (2 ml) was administered with aseptic measures. To prevent any inconsistency, all shots were administered by the same anesthesiologist. Each patient was placed in a prone position. A spinal needle of length 3.5 inches, gauge 23, was carefully advanced under fluoroscopic (real-time X-ray) guidance towards the oblique view of the safe triangle, which is formed by the lateral border of the vertebral body, the pedicle, which forms the roof of the triangle, and a tangential base corresponding to the nerve root exiting. Fluoroscopic projections of the anteroposterior and medial rectus muscles confirmed the needle's proper positioning.

After field preparation and 1% lidocaine skin anaesthetic, the facet joints were pierced with spinal needles (23 G) while being observed under fluoroscopic monitoring. One ml of crystalline triamcinolone acetate (50 mg) and 2 ml of lidocaine (1% each) were applied to one joint in one group and 1 ml of dexamethasone (8 mg) and 2 ml of lidocaine in another group.

The IA injections were started with mild pressure that increased joint capsule tension. We cautiously discontinued periarticular injections when the joint cavity became sufficiently full to prevent the joint capsule from rupturing.

Following IA injection, patients were followed up at baseline, one month, three months, and six months. At the time of follow-up, each patient was assessed by using VAS, MODI, and the short-form McGill Pain Questionnaire.

VAS

A unidimensional measure of the intensity of pain in adult populations is the VAS. It is a continuous scale. The scoring ranges from 0 to 10, where a score of 0 implies no pain, and a score of 10 implies worst pain [14].

Oswestry low back pain disability index

Investigators and disability assessors used the Oswestry Disability Index (ODI), as a crucial instrument to determine the persistent functional handicap of a patient. The exam has been available since 1980 and is regarded as the gold standard for functional outcome measures for low back pain. There are five points for each question; the first answer receives zero, the second receives one, and so on. After adding up all ten questions, assigning of score is done [15].

McGill Pain Questionnaire

It is a multifaceted way to quantify how much discomfort adults with chronic pain perceive. Patients employ three major groups of word descriptors, sensory, emotional, and evaluative, in the McGill Pain Questionnaire to describe their subjective pain experiences. To find out the characteristics of the pain experience, it also includes an intensity scale and other things. The purpose of the questionnaire was to get quantitative data about clinical pain that could be statistically analyzed [16].

Statistical analysis

Data obtained was entered into MS Excel (Microsoft Corporation, Redmond, Washington, United States), and data analysis was done using IBM SPSS Statistics for Windows, Version 26, (Released 2019; IBM Corp., Armonk, New York, United States). Gender and pain characteristics, such as duration of pain and treatment of single and multiple levels, were expressed in frequencies and percentages. Age and other scoring parameters were expressed in mean and standard deviation. A comparison of the effect of injections between the groups was done by using an unpaired student’s T-test. A p-value less than 0.05 was considered statistically significant.

## Results

A total of 60 participants were analyzed for results. Group A consisted of 27 participants and Group B comprised 33 participants. Socio-demographic details of the participants are given in Table 1. The mean age of the participants in Group A was 46.77 years, whereas it was 49.30 years in Group B. Both groups predominantly comprised males. Many participants (43.3% in Group A and 36.7% in Group B) had pain for around four months.

**Table 1:** Distribution of sociodemographic variables and pain characteristics among the participants (n=60)

Variable	Groups	
	Group A (n=27)	Group B (n=33)
Age	46.77 ± 4.92	49.30 ± 5.29
Gender		
Male	24 (45.2%)	29 (54.8%)
Female	3 (42.8%)	4 (57.2%)
Duration of pain		
Three months	6 (50%)	6 (50%)
Four months	10 (41.6%)	14 (58.4%)
Five months	7 (46.6%)	8 (53.4%)
Six months	4 (44.4%)	5 (55.6%)
Levels treated		
Single	25 (46.2%)	29 (53.8%)
Multiple	2 (33.3%)	4 (66.4%)

The distribution of the VAS scores among the two groups is given in Table 2. On applying the independent T-test, there was a significant difference in pain scores between the two groups before and after the intervention at all intervals (the p-value was less than 0.05). Group B, which received triamcinolone acetate, reported low pain when compared to the other group.

**Table 2:** Distribution of Visual Analog Scale (VAS) scoring among the participants (n=60)

VAS scoring time interval	Group A (n=27)	Group B (n=33)	p-value
Pre-procedure	7.41 ± 0.73	6.23 ± 0.56	0.030
Day one	3.24 ± 0.57	5.17 ± 0.83	0.000
One month	2.79 ± 0.41	3.13 ± 0.34	0.001
Three months	3.31 ± 0.60	2.73 ± 0.45	0.000
Six months	4.41 ± 0.68	2.33 ± 0.47	0.000

The distribution of ODI scoring among the two groups is given in (Table 3). There was a significant difference between the two groups at one month (p value=0.001), three months (p value=0.005) and 6 months (p value=0.000) after the procedure, whereas on day one of post procedure there was no significant difference between the groups (p value was more than 0.05).

**Table 3:** Distribution of the Oswestry low back pain Disability Index (ODI) scoring among the participants

ODI scoring time interval	Group A (n=27)	Group B (n=33)	p-value
Pre-procedure	20.17 ± 2.13	19.53 ± 1.47	0.186
Day one	9.59 ± 2.21	10.57 ± 1.87	0.071
One month	8.14 ± 1.52	7.43 ± 0.81	0.001
Three months	7.03 ± 1.47	6.50 ± 0.73	0.005
Six months	17.62 ± 2.21	6.40 ± 0.67	0.000

The distribution of McGill Pain scoring among the two groups is given in Table 4. There was no significant difference in pain scores between the two groups before the intervention by applying the independent T-test. There was a significant difference in pain score between the two groups post-intervention at one, three, and six months (p-value=0.000 at three months). On day one and at one month, Group A, which received dexamethasone, was found to have low pain scores, whereas at three and six months, Group B, which received triamcinolone, had low pain scores.

**Table 4:** Distribution of McGill Pain Questionnaire scoring among the participants (n=60)

Mc Gill Pain Questionnaire scoring time interval	Group A (n=27)	Group B (n=33)	p-value
Pre-procedure	58 ± 3.95	59.23 ± 3.75	0.224
Day one	55.17 ± 1.77	64.90 ± 1.88	0.000
One month	56.17 ± 1.60	63.17 ± 2.06	0.000
Three months	62.48 ± 2.73	59.17 ± 2.26	0.000
Six months	65 ± 2.10	54.10 ± 6.67	0.000

## Discussion

In our study, there was a significant difference between both the groups after the procedure, showing the effectiveness of triamcinolone acetate. In a trial done in Germany in 2013, which included patients who had radiofrequency denervation of L3/L4-L5/S1 segments and patients who got IA steroid infiltrations in the LFJs (L3/L4-L5/S1). The Roland-Morris Questionnaire served as the primary outcome measure. The ODI and the VAS served as secondary end goals. At baseline and after six months, every outcome evaluation was completed. Nearly 56 patients were randomly assigned. Both groups saw less pain and an increase in their functional status. There were no significant differences between primary endpoints and secondary endpoints [1].

When compared to patients who had transforaminal injections, those who had facet injections reported much lower pain scores. Facet injections are therefore a reliable and risk-free substitute for transforaminal injections to treat cervical radiculopathy [17]. In a review by Benzon et al., the obstruction of the blood arteries supplying the spinal cord and brain by the particulate steroid was attributed to central nervous system damage following transforaminal epidural steroid injections [18]. Comparisons were made between different steroids to identify the steroid with larger particles. There were no discernible particles in the pure liquids of dexamethasone sodium phosphate and betamethasone sodium phosphate. Triamcinolone acetonide had an opaque, amorphous appearance. The compound betamethasone was opaque and amorphous. Dexamethasone and triamcinolone were found to have a smaller proportion of larger particles when compared to other steroids.

In a study by Tiso et al. in 2004 in North America, after the lumbar transforaminal block, persistent paraplegia was documented [19]. The possibility of an undetected IA injection was raised. So, the study advised precautions that should be taken to prevent precipitation and aggregate clumping in the neural foramina. The study iterated using corticosteroid solutions, such as betamethasone and dexamethasone sodium phosphate. Because of the smaller size of the particulate matter, suspension could be avoided, but betamethasone sodium phosphate-betamethasone acetate (BSP-BA) would be preferred if a suspension were to be used. The usage of dexamethasone sodium phosphate alone may be wise to consider because of the risk of contamination [19].

In a similar study by Madavi et al. in 2020 in Maharashtra, India, among 66 patients, by randomizing 33 patients in dexamethasone and triamcinolone groups, statistically significant differences were found in the ODI (18.67 ± 7.13 in the triamcinolone group, 35.83 ± 5.10 in the dexamethasone group), McGill Pain Questionnaire (3.73 ± 1.15 in the triamcinolone group, 6.55 ± 0.51 in the dexamethasone group), and VAS score (2.85 ± 0.83 in the triamcinolone group, 5.76 ± 0.75 in the dexamethasone group), but it was more pronounced in the triamcinolone group [20].

In a study by Shakir et al. among 441 patients to test the efficacy of dexamethasone and triamcinolone, 220 participants had injections of 40 mg of triamcinolone, with a mean reduction in pain score of 2.33 on a scale of 10 points [21]. The remaining 221 participants received dexamethasone (15 mg per injection), with a mean reduction in pain score of 2.38 points on a scale of 10 points. According to the results of the two-sample F-test for variance, there was no significant difference in the variance of these two groups. The mean reduction in pain score between the two groups did not differ statistically significantly, according to the two-sample t-test with comparable variance.

In a longitudinal cohort study by McCormick et al. in 2015 in Chicago, in 78.8% (1235) and 21.2% (333) of the individuals, betamethasone and triamcinolone were used, respectively [22]. A significantly higher percentage of patients who received triamcinolone (44.4%) reported pain reduction of more than 50% at a short-term follow-up of four weeks, compared to 26.8% of patients who received betamethasone.

A study by Yachouchi et al. in 2013 in the United States was done to determine if dexamethasone (10 mg) was less clinically efficacious than triamcinolone (80 mg) and betamethasone (12 mg) when administered as lumbar TFESIs to patients with radicular discomfort with or without radiculopathy [23]. Prior to TFESI, as well as at the two-week and two-month follow-ups, subjects were evaluated using a pain numerical rating scale and Roland-Morri’s disability questionnaire. Dexamethasone was superior to particulate steroids in both pain alleviation and functional improvement at two months, using continuous outcomes. This study contrasts our results, but the difference may be because of the usage of different parameters and study settings.

A systematic review by Mehta et al. in 2017 in New York, United States, also suggested that nonparticulate steroids such as dexamethasone should be preferred as first-line drugs in epidural steroid injections [10]. The authors advised using nonparticulate steroids for lumbar TFESI in patients with lumbar radicular pain because particulate and nonparticulate steroids were equally effective in reducing pain and improving function in patients with lumbar radiculopathy caused by stenosis or disk herniation.

Limitations

The study's findings may not be generalizable to all populations as the study was carried out at a single institution. This limits the broader applicability of the results. There may be confounding variables that were not adequately controlled for in the analysis. Factors such as age, comorbidities, severity of facet arthropathy, concurrent treatments, and patient compliance could influence the outcomes but might not have been fully accounted for in the study. The duration of follow-up might be limited, potentially missing long-term outcomes or a recurrence of symptoms beyond the study period. This could affect the assessment of the sustained efficacy of the IA steroid injections.

## Conclusions

When comparing the two drugs, dexamethasone and triamcinolone acetate, pain relief was more in the group that received triamcinolone acetate and there were no reports of complications with both the drugs. When a lumbar TFESI is used to treat radiculopathy in the lumbar area, particulate medication (triamcinolone acetate) is more effective than nonparticulate medication (dexamethasone).
